# Daytime Sleepiness and Quality of Life in Obstructive Sleep Apnoea Patients before and after Long-Term Mandibular Advancement Device Treatment

**DOI:** 10.3390/dj10120226

**Published:** 2022-11-30

**Authors:** Signe Halfeld, Liselotte Sonnesen

**Affiliations:** Section for Orthodontics, Department of Odontology, Faculty of Health and Medical Sciences, University of Copenhagen, 2200 Copenhagen, Denmark

**Keywords:** sleep-disordered breathing, mandibular advancement treatment, quality of life, daytime sleepiness

## Abstract

This study compared daytime sleepiness and quality of life in OSA patients with healthy controls and compared sleepiness and quality of life in OSA patients before and after long-term treatment with a mandibular advancement device (MAD). A total of 27 OSA patients (18 men, 9 women, mean age 52.3 years) and 32 healthy age- and sex-matched controls (20 men, 12 women, mean age 51.1 years) were included. At baseline and after MAD treatment, daytime sleepiness and quality of life were recorded by the Epworth Sleepiness Scale (ESS) and Short Form-36 questionnaires (SF-36). Daytime sleepiness occurred significantly more often in OSA patients compared to controls at baseline (*p* = 0.01). The quality of life domains Energy and vitality (*p* < 0.0001), General perception of health (*p* = 0.0002), Mental health (*p* = 0.0031), Social functioning (*p* = 0.0119), Role limitations due to emotional problems (*p* = 0.0173) and Physical functioning (*p* = 0.0226) were significantly poorer in OSA patients compared to controls at baseline. After long-term MAD treatment, daytime sleepiness decreased (*p* < 0.01) and the quality of life domain Energy and Vitality increased (*p* < 0.01) in OSA patients compared to baseline. The results of the present study support the relevance of MAD treatment as an effective tool for decreasing daytime sleepiness and increasing the quality of life in OSA patients—also in the long term.

## 1. Introduction

Obstructive sleep apnoea (OSA) is the most common sleep-related breathing disorder [1]. OSA is characterised by episodes of collapse of the upper airways during sleep, which causes cessation or reduction of airflow in spite of continued breathing attempts [2]. This leads to reduced oxygen saturation (hypoxia) and elevated levels of carbon dioxide in the bloodstream (hypercapnia). The autonomic nervous system is activated, the patient briefly awakens (arousal) and sleep is interrupted [3,4]. 

The most significant symptoms in adult OSA patients are increased daytime sleepiness [5,6,7] and loud snoring [8]. Patients with untreated OSA show increased morbidity with regard to a number of disorders in comparison with the general population [4]. For instance, hypertension, cardiovascular disorders and type 2 diabetes are overrepresented in untreated OSA patients [3,9,10]. In addition, untreated OSA patients generally have increased levels of sickness absence, which has a significant socio-economic impact while also stressing the patient [11].

In recent years, the social and psychological consequences of OSA have received more attention. OSA has been associated with social problems and traffic accidents due to a lack of rest, daytime sleepiness and involuntary sleep episodes [11,12,13]. Studies have also shown that OSA patients have a decreased quality of life in comparison with healthy subjects [14,15,16].

Treatment of OSA with Continuous Positive Airway Pressure (CPAP) is considered the “golden standard” [3]. However, CPAP can be difficult to get accustomed to, and for some patients proves to be an impossible task [17]. Side effects such as frequent colds and skin irritation are often reported [18]. Inconveniences such as noise and problems when travelling have also been reported [17]. For social snoring, mild to moderate OSA or severe OSA where CPAP is not an option, treatment with a mandibular advancement device (MAD) may be considered [18,19,20,21,22]. MAD is a one- or two-piece appliance that works by protruding the mandible during sleep, which helps prevent collapse of the upper airway. Thus, the appliance is an active orthodontic appliance. Diagnosis before MAD treatment and repeated individual controls and adjustment (titration) of the MAD appliance by a specialist in orthodontics or a special trained dentist are essential in order to obtain treatment success and minimize side effects [21,23]. Due to the complexity of OSA, treatment of OSA with a MAD should be performed by an interdisciplinary team. The initial diagnosis of OSA must be performed by a sleep physician, and when indicated, in combination with other medical doctor specialists before referral to a specialist in orthodontics or a specially trained dentist [20,21,23]. The oro-craniofacial diagnosis performed be the dental specialist prior to MAD treatment should include an assessment of the dentition, dental occlusion, craniofacial morphology and masticatory muscles and temporomandibular joints [20,21,23]. The oro-craniofacial diagnosis is essential for excluding patients at high risk of unwanted side effects or patients where MAD treatment is contraindicated [20,21,23,24].

MAD treatment can reduce the incidence of apnoea by up to 80% in patients with mild to moderate OSA [18,19,21]. When MAD treatment is carried out by qualified and experienced dentists with regular follow-ups, MAD can be used long-term [4,20,21,25,26,27,28,29]. Short-term MAD treatment can also improve quality of life and reduce daytime sleepiness in OSA patients [19,30,31,32]. It seems that no studies have so far been conducted on how long-term MAD treatment affects daytime sleepiness and quality of life.

The aims of the present study were to examine daytime sleepiness and quality of life in patients with OSA in comparison with healthy controls and to compare sleepiness and quality of life in OSA patients before and after long-term MAD treatment.

## 2. Materials and Methods

### 2.1. Subjects

#### 2.1.1. OSA Patients

The data on the OSA patient group were gathered as a part of another study from the Dental Sleep Clinic, Section for Orthodontics, Department of Odontology at the University of Copenhagen. The OSA group consisted of 71 subjects who were diagnosed with OSA by ear–nose–throat medical doctors using polygraphy (Embletta, TK2) and who were subsequently referred for treatment at the Dental Sleep Clinic in the period of 2011–2013.

The OSA group had the following inclusion criteria:

- AHI > 5
- Minimum of 20 teeth, including the two first lower molars
- Bone loss <50%
- Evenly distributed teeth in occlusal contact
- Filled-in Epworth questionnaire on daytime sleepiness and SF-36 questionnaire on quality of life at treatment start
- Lateral cephalograms at treatment start

The exclusion criteria were:

- AHI < 5
- Severe somatic and/or mental disease
- Severe cardiovascular disease
- Chronic pronounced nasal stenosis
- Hypertrophic tonsils
- Symptoms from TMJ and/or masticatory muscles that required treatment before MAD-treatment
- Open bite malocclusion
- Angle class 2 div.2
- Severe craniofacial deformities
- Facial wear of lower incisors
- Jaw protrusion <6 mm
- Insufficiently filled-in questionnaires
- Missing lateral cephalograms

About half of the OSA patients were treated with drugs for various medical conditions. 

The MAD treatment of the OSA patients was performed by the same Orthodontics specialist. The manufactured mandibular advancement device (MAD) was a custom-made, removable, two-piece, adjustable device. The mean mandibular protrusion was 76.9% and the average treatment time between T1 and T2 was 14.6 months, ranging between 8–32 months.

**Figure 1:**
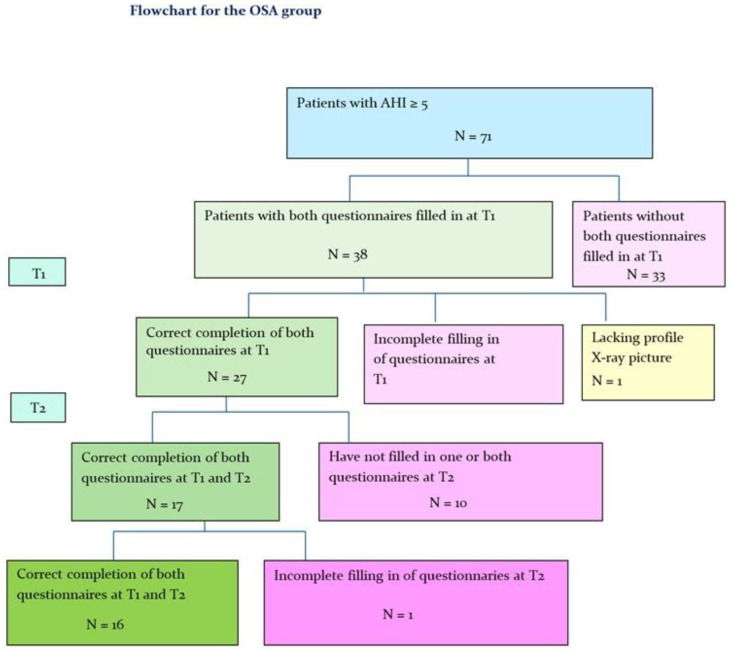
Flowchart for the OSA group. T1: Before treatment with MAD; T2: After treatment with MAD.

In total, 27 patients, 18 men, mean age 49.8 years (27–68 years) and 9 women, mean age 54.8 years (26–73 years)) out of the 71 patients in the OSA group met the inclusion criteria (Figure 1). The mean AHI at T1 was 22 (7–57) for men and 17.1 for women (8–35).

After MAD treatment (T2) 16 patients, 9 men, mean age 51.1 years (39–61 years) and 7 women, mean age 51.1 years (39–61 years)) remained in the OSA group (Figure 1).

#### 2.1.2. Control Group

The control group consisted of 32 employees at the Department of Odontology, University of Copenhagen. The control group was matched with the OSA group by sex and age and met the following inclusion criteria:

- Employed between June 2015 and April 2016
- No known OSA
- Neutral or minor malocclusion traits that did not require orthodontic treatment

Exclusion criteria:

- Known OSA
- Severe malocclusion traits or craniofacial anomalies

The control group consisted of 12 women and 20 men. The mean age was 49.1 years for men (25–68 years) and 53.1 years for women (28–74 years).

The project was approved by the Danish Data Protection Agency (data: J.no.2012-54-0041) and the Danish National Committee for Health Research Ethics (ref.no.H-3-2011-086).

### 2.2. Methods

Daytime sleepiness and quality of life were registered before and after MAD treatment (T1 and T2, respectively).

#### 2.2.1. Daytime Sleepiness

Daytime sleepiness was measured using the Epworth Sleepiness Scale questionnaire (ESS) [33]. The questionnaire consists of 8 questions to measure daytime sleepiness [5]. Subjects were asked to state their risk of falling asleep in 8 different situations on a scale from 0 to 3. The higher the score, the higher the daytime sleepiness, with 24 as the highest score.

#### 2.2.2. Quality of Life

The subjects’ quality of life was measured using the SF-36 questionnaire [34]. The questionnaire has previously been validated in the Danish language [35]. The questionnaire provides a general health assessment within 8 health domains (physical functioning, social functioning, role limitations due to physical functions, role limitations due to emotional problems, mental health, energy and vitality, pain and general perception of health). There are a total of 36 questions scored from 0–100, where 100 represents the highest quality of life.

### 2.3. Statistical Analysis

The statistical analysis was carried out using the statistics software SAS (Statistical Analysis Software Version 9.4, Cary, NC, USA). The results were considered significant at *p*-values <0.05. The differences in daytime sleepiness and quality of life between the OSA group and the control group were analysed using general linear regression models adjusted for age and sex. Daytime sleepiness and quality of life were dependent variables in the models, while the OSA and control groups were independent variables. The differences are shown with 95% confidence intervals, *p*-values and the coefficient of determination (r^2^).

The differences in daytime sleepiness and quality of life before and after treatment (T2–T1) were analysed using general linear regression models adjusted for age and sex. The differences are shown with 95% confidence intervals, *p*-values and the correlation coefficient (r).

## 3. Results

**Figure 2:**
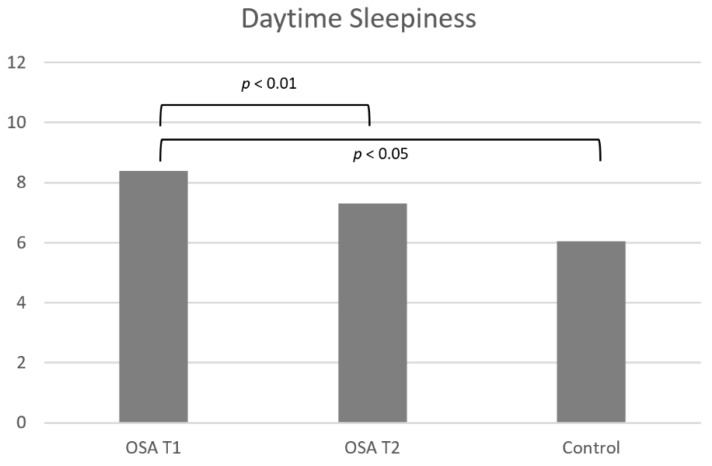
Difference in daytime sleepiness between the OSA group and the control group before MAD treatment (T1) and before (T1) and after (T2) MAD treatment in the OSA group. Significant differences are shown with lines and *p* values.

**Figure 3:**
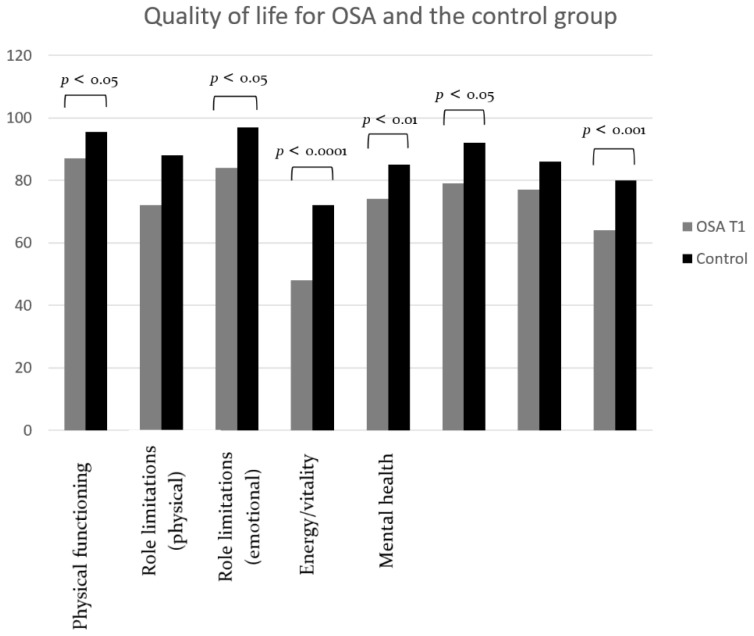
Differences in quality of life between the OSA group and the control group before (T1) MAD treatment. Significant differences are shown with lines and *p* values.

The difference between daytime sleepiness and quality of life between the OSA group and the control group at T1 is shown in Table 1 and Figure 2 and Figure 3. 

Daytime sleepiness was significantly larger for the OSA group compared to the control group (*p* = 0.0110; r^2^ = 0.16; Table 1, Figure 2).

Six quality of life domains were significantly lower for the OSA group compared to the control group (Table 1, Figure 3), meaning that quality of life was significantly decreased for the OSA group in these domains. Quality of life for the domains Energy and vitality (*p* < 0.0001; r^2^ = 0.33), General perception of health (*p* = 0.0002; r^2^ = 0.25), Mental health (*p* = 0.0031; r^2^ = 0.22), Social functioning (*p* = 0.0119; r^2^ = 0.19), Role limitations due to emotional problems (*p* = 0.0173; r^2^ = 0.13) and Physical functioning (*p* = 0.0226; r^2^ = 0.10) was significantly decreased in the OSA group in comparison with the control group.

**Figure 4:**
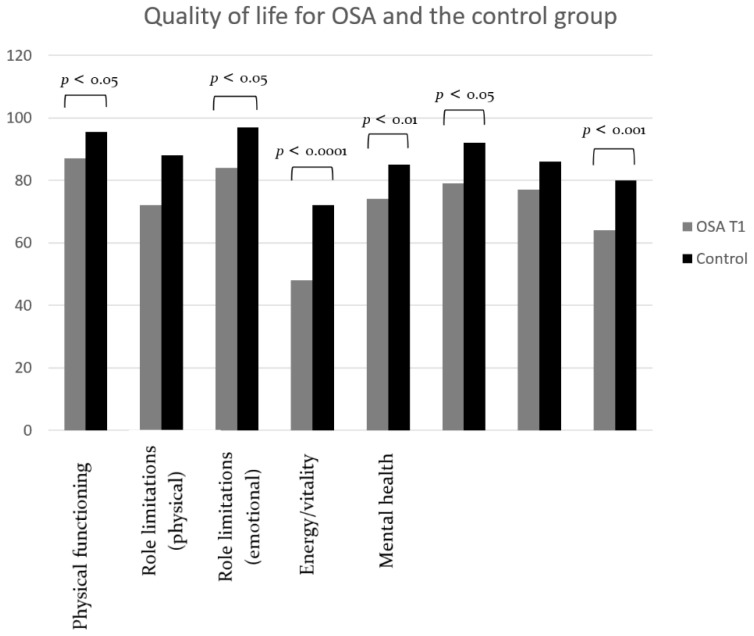
Differences in quality of life for the OSA group before (T1) and after (T2) MAD treatment. Significant differences are shown with lines and *p* values.

The difference in daytime sleepiness and quality of life for the OSA group before and after long-term MAD treatment is shown in Table 2 and Figure 2 and Figure 4. 

The OSA group experienced significantly less daytime sleepiness after MAD treatment than before treatment (*p* = 0.0018; r = 0.85; Table 2 and Figure 2).

With regard to quality of life, the Energy/vitality domain (SF36d, *p* = 0.0037; r = 0.47; Table 2, Figure 4) was significantly increased after MAD treatment compared to before. In addition, there was a tendency for an increase in the Social functioning domain (SF36f, *p* = 0.05; r = 0.54; Table 2, Figure 4) after MAD treatment, but the difference was not significant.

## 4. Discussion

This study aimed to compare daytime sleepiness and quality of life between OSA patients and healthy controls. Furthermore, the present study focused on sleepiness and life quality in patients with OSA prior to and after long-term MAD treatment. Previously, it has been shown that OSA patients have lower life-quality in comparison with healthy subjects [14,15,16] and that MAD treatment can improve life quality and reduce sleepiness in OSA patients [19,30,31,32] in the short term. How long-term MAD treatment affects sleepiness and life quality has not previously been reported in the literature. 

In the present study, the OSA patients were all diagnosed with an AHI >5 using polygraphy and the sex ratio was 2/3 male and 1/3 female, where the mean age was 49.8 years for men and 54.8 years for women. Previous studies have shown that OSA occurs more frequently in men than women, with a ratio of 2:1 [1,36]. Furthermore, it is well-known that OSA occurs earlier and more often in men, and for women more often after the menopause [4,36,37]. Thus, the OSA patients in the present study were all well-diagnosed prior to MAD treatment and represent the adult OSA population in general. In the present study, a limitation may be that in about half of the patients, one or both questionnaires were incompletely filled out at the follow-up time point (T2). The incomplete questionnaires occurred randomly because of administrative reasons. Thus, this may not be considered a bias. The questionnaires in the present study were recognized, validated standard questionnaires [15,33,38,39,40].

It is well documented that OSA is associated with increased daytime sleepiness in adults [5,6,7]. The present study confirmed this by the significant difference in daytime sleepiness between the OSA group and the control group. The difference in daytime sleepiness between the OSA group and the control group could be caused by the impact that OSA has on patients’ sleep. Previously, it has been shown that OSA patients have reduced deep sleep, i.e., stage 3 NREM sleep and REM sleep [4,41]. It has also been shown that fragmented NREM sleep in patients with breathing disorders during sleep is associated with increased daytime sleepiness [42].

In the present study in which patients with OSA and healthy controls were compared, the OSA patients showed significantly lower life-quality for the domains Physical functioning, Role limitations due to emotional problems, Energy and vitality, Mental health, Social functioning and General perception of health. Previous studies have disagreed on how OSA may be associated with quality of life. Baldwin et al. 2001 showed that patients with OSA only had decreased life-quality within the Vitality and energy domain [43]. However, Gall et al. 1993 found that patients with OSA had decreased life-quality within the Social functioning, Role limitations due to physical functions, Physical functioning, Role limitations due to emotional problems, Vitality and energy as well as Mental health domains [14]. Such differences in results may be caused by the various study designs and study samples used, as well as differences in sex balance and OSA severity. Furthermore, quality of life is a complex term that is affected by various factors such as, e.g., depression, obesity and illness [44,45]. 

In the present study, the OSA group was significantly less tired after long-term MAD treatment (mean MAD treatment time of 14.6 months). It has previously been shown that short-term MAD treatment reduces daytime sleepiness in OSA patients [19,30,32,46]. However, Marklund et al. 2015 found no difference in daytime sleepiness in subjects who snored or suffered from mild/moderate OSA after 4 months of MAD treatment [47]. The difference may be because Marklund examined subjects with a smaller AHI and for a shorter observation period [47] than in the present study.

The OSA patients in the present study showed a significant improvement in their quality of life within the domain Vitality and energy after a mean MAD treatment time of 14.6 months. This is in agreement with previous studies of OSA patients after short-term MAD treatment. Petri et al. 2008 showed an effect on patients with moderate to severe OSA in the quality of life Vitality and energy domain after a 4-week period of MAD treatment [19]. Barnes et al. 2004 examined 80 OSA patients with mild to moderate OSA and found that quality of life improved after a 3-month period of MAD treatment [48]. However, other studies showed no significant difference in quality of life before and after a 3-month period of MAD treatment for OSA patients [46,49]. One study showed improvement in the quality of life after 24 months of MAD treatment for OSA patients [32], but only the general quality of life score was used instead of specific quality of life domains used in the present study. Thus, the Vitality and energy domain appears to be the most relevant domain related to adult OSA patients undergoing MAD treatment [19,39], which is in agreement with the present study of the long-term MAD treatment of adult OSA patients.

## 5. Conclusions

The present study found significantly increased daytime sleepiness and significantly decreased quality of life within the domains of Energy and vitality, General perception of health, Mental health, Social functioning, Role limitations due to emotional problems and Physical functioning in OSA patients in comparison with healthy control subjects, which confirms the results of previous studies.

Furthermore, the present study found significantly decreased daytime sleepiness and increased quality of life within the Energy and vitality domain in OSA patients after long-term MAD treatment compared to before MAD treatment. These new results on the long-term MAD treatment of OSA patients found in the present study may prove valuable in treatment considerations for OSA patients and can be included in future discussions regarding the MAD treatment of OSA patients. 

## Figures and Tables

**Table 1:** Differences in daytime sleepiness and quality of life between the OSA group and the control group before MAD treatment. The estimates are shown with 95% confidence intervals, *p*-values and coefficients of determination (r^2^). Adjusted for sex and age.

Variables	Difference OSA vs. Control (95% Confidence Interval)	r^2^ (Coefficient of Determination)	*p*-Value
Daytime sleepiness			
ESS	2.36 (0.56−4.15)	0.16	0.0110 *
Quality of life			
Physical functioning	−7.67 (−14.23–(−1.12))	0.10	0.0226 *
Role limitations (physical)	−16.32 (33.58–0.94)	0.06	0.06
Role limitations (emotional)	−14.57 (−26.46–(−2.68))	0.13	0.0173 *
Energy/vitality	−24.51 (−34.44–(−14.58))	0.33	<0.0001 ****
Mental health	−11.72 (−19.31–(−4.13))	0.22	0.0031 **
Social functioning	−13.72 (−24.28–(−3.15))	0.19	0.0119 *
Pain	−8.08 (−20.12–3.96)	0.04	0.18
General health perceptions	−16.29 (−24.54–(−8.04))	0.25	0.0002 ***

ESS: Epworth Sleepiness Scale * *p* < 0.05 ** *p* < 0.01 *** *p* < 0.001 **** *p* < 0.0001.

**Table 2:** Differences in daytime sleepiness and quality of life in the OSA group before (T1) and after (T2) MAD treatment. The estimates are shown with 95% confidence intervals, *p*-values and correlation coefficients (r). Adjusted for sex and age.

Variables	Difference T2 vs. T1 (95% Confidence Interval)	r (Correlation Coefficient)	*p*-Value
Daytime sleepiness			
ESS	−2.25 (−3.52−(−0.98))	0.85	0.0018 *
Quality of life			
Physical functioning	−2.19 (−6.75–2.37)	0.84	0.32
Role limitations (physical)	7.81 (−11.47–27.10)	0.56	0.40
Role limitations (emotional)	6.26 (−11.17–23.69)	0.56	0.46
Energy/vitality	18.13 (6.87–28.38)	0.47	0.0037 *
Mental health	4.75 (−7.97–17.47)	0.04	0.44
Social functioning	10.84 (−0.17–22.05)	0.54	0.05
Pain	7.50 (−7.27–22.27)	0.30	0.30
General health perceptions	4.38 (−1.45–10.20)	0.85	0.13

ESS: Epworth Sleepiness Scale: * *p* < 0.01.

## Data Availability

The data presented in this study are available on request from the corresponding author. The data are not publicly available due to privacy.

## References

[1] Løvschall C., Søbjerg L.M., Nørregaard O., Prætorius C., Hilberg O., Jennum P., Tipsmark L.S., Pedersen T.V., Nielsen C.P. (2013). Medicinsk Teknologi Vurdering af Obstruktiv Søvnapnø [Technological Medical Assessment of Obstructive Sleep Apnoea].

[2] Guilleminault C., Tilkian A., Dement W.C. (1976). The Sleep Apnea Syndromes. Annu. Rev. Med..

[3] Balachandran J.S., Patel S.R., Cotton D., Rao J.K., Taichman D., Williams S. (2014). In the Clinic: Obstructive Sleep Apnea. Ann. Intern. Med..

[4] Jordan A.S., McSharry D.G., Malhotra A. (2014). Adult obstructive sleep apnoea. Lancet.

[5] Johns M.W. (1993). Daytime Sleepiness, Snoring, and Obstructive Sleep Apnea: The Epworth Sleepiness Scale. Chest.

[6] Gottlieb D.J., Whitney C.W., Bonekat W.H., Iber C., James G.D., Lebowitz M., Nieto J., Rosenberg C.E. (1999). Relation of Sleepiness to Respiratory Disturbance Index: The Sleep Heart Health Study. Am. J. Respir. Crit. Care Med..

[7] Ohayon M.M. (2012). Determining the level of sleepiness in the American population and its correlates. J. Psychiatr. Res..

[8] Sunitha C., Aravindkumar S. (2009). Obstructive sleep apnea: Clinical and diagnostic features. Ind. J. Dent Res..

[9] Gonzaga C., Bertolami A., Bertolami M., Amodeo C., Calhoun D. (2015). Obstructive sleep apnea, hypertension and cardiovascular diseases. J. Hum. Hypertens..

[10] Tahrani A.A., Ali A., Raymond N.T., Begum S., Dubb K., Altaf Q.A., Piya M.K., Barnett A.H., Stevens M.J. (2013). Obstructive Sleep Apnea and Diabetic Nephropathy: A cohort study. Diabetes Care.

[11] Jennum P., Kjellberg J. (2011). Health, social and economical consequences of sleep-disordered breathing: A controlled national study. Thorax.

[12] Ward K.L., Hillman D.R., James A., Bremner A.P., Simpson L., Cooper M.N., Palmer L.J., Fedson A.C., Mukherjee S. (2013). Excessive Daytime Sleepiness Increases the Risk of Motor Vehicle Crash in Obstructive Sleep Apnea. J. Clin. Sleep Med..

[13] Hirsch Allen M.A.J., Bansback N., Ayas N.T. (2015). The Effect of OSA on Work Disability and Work-Related Injuries. Chest.

[14] Gall R., Isaac L., Kryger M. (1993). Quality of Life in Mild Obstructive Sleep Apnea. Sleep.

[15] Moyer C.A., Sonnad S.S., Garetz S.L., Helman J.I., Chervin R.D. (2001). Quality of life in obstructive sleep apnea: A systematic review of the literature. Sleep Med..

[16] Yang E.H., Hla K.M., McHorney C.A., Havighurst T., Badr M.S., Weber S. (2000). Sleep apnea and quality of life. Sleep.

[17] Shapiro G.K., Shapiro C.M. (2010). Factors that influence CPAP adherence: An overview. Sleep Breath.

[18] Hoffstein V. (2007). Review of oral appliances for treatment of sleep-disordered breathing. Sleep Breath.

[19] Petri N., Svanholt P., Solow B., Wildschiødtz G., Winkel P. (2008). Mandibular advancement appliance for obstructive sleep apnoea: Results of a randomised placebo controlled trial using parallel group design. J. Sleep Res..

[20] Levrini L., Sacchi F., Milano F., Polimeni A., Cozza P., Bernkopf E., Segù M., Zucconi M., Vicini C., Brunello E. (2016). Italian recommendations on dental support in the treatment of adult obstructive sleep apnea syndrome (OSAS). Ann. Stomatol..

[21] Knappe S.W., Sonnesen L. (2018). Mandibular positioning techniques to improve sleep quality in patients with obstructive sleep apnea: Current perspectives. Nat. Sci. Sleep.

[22] Petri N., Christensen I.J., Svanholt P., Sonnesen L., Wildschiødtz G., Berg S. (2019). Mandibular advancement device therapy for obstructive sleep apnea: A prospective study on predictors of treatment success. Sleep Med..

[23] Knappe S.W., Bakke M., Svanholt P., Petersson A., Sonnesen L. (2017). Long-term side effects on the temporomandibular joints and oro-facial function in patients with obstructive sleep apnoea treated with a mandibular advancement device. J. Oral. Rehabil..

[24] Svanholt P., Petri N., Wildschiødtz G., Sonnesen L. (2015). Influence of craniofacial and upper spine morphology on mandibular advancement treatment in patients with sleep apnoea: A pilot study. Eur. J. Orthod..

[25] Epstein L.J., Kristo D., Strollo P.J., Friedmann N., Malhotra A., Patil S.P., Ramar K., Rogers R., Schwab R.J., Weaver E.M. (2009). Clinical guideline for the evaluation, management and long-term care of obstructive sleep apnea in adults. J. Clin. Sleep Med..

[26] Sharples L.D., Clutterbuck-James A.L., Glover M.J., Bennett M.S., Chadwick R., Pittman M.A., Quinnell T.G. (2016). Meta-analysis of andomized controlled trials of oral mandibular advancement devices and continuous positive airway pressure for obstructive sleep apnoea-hypopnoea. Sleep Med. Rev..

[27] Sutherland K., Cistulli P. (2011). Mandibular advancement splints for the treatment of sleep apnea syndrome. Swiss Med. Wkly.

[28] Ramar K., Dort L.C., Katz S.G., Lettieri C.J., Harrod C.G., Thomas S.M., Chervin R.D. (2015). Clinical Practice Guideline for the Treatment of Obstructive Sleep Apnea and Snoring with Oral Appliance Therapy: An Update for 2015. J. Clin. Sleep Med..

[29] Fagundes N.C., Minervini G., Alonso B.F., Nucci L., Grassia V., d’Apuzzo F., Puigdollers A., Perillo L., Flores-Mir C. (2022). Patient-reported outcomes while managing obstructive sleep apnea with oral appliances: A scoping review. J. Evid Base Dent Pract..

[30] Gotsopoulos H., Chen C., Qian J., Cistulli P.A. (2002). Oral appliance therapy improves symptoms in obstructive sleep apnea: A randomized, controlled trial. Am. J. Respir. Crit. Care Med..

[31] Serra-Torres S., Bellot-Arcís C., Montiel-Company J.M., Marco-Algarra J., Almerich-Silla J.M. (2016). Effectiveness of Mandibular Advancement Appliances in Treating Obstructive Sleep Apnea Syndrome: A Systematic Review. Laryngoscope.

[32] Ghazal A., Sorichter S., Jonas I., Rose E.C. (2009). A randomized prospective long-term study of two oral appliances for sleep apnoea treatment. J. Sleep Res..

[33] Johns M.W. (1991). A New Method for Measuring Daytime Sleepiness: The Epworth Sleepiness Scale. Sleep.

[34] Jenkinson C. (1998). The SF-36 physical and mental health summary measures: An example of how to interpret scores. J. Health Serv. Res. Policy.

[35] Bjorner J.B., Thunedborg K., Kristensen T.S., Modvig J., Bech P. (1998). The Danish SF-36 Health Survey: Translation and preliminary validity studies. J. Clin. Epidemiol..

[36] (1999). Sleep-Related Breathing Disorders in Adults: Recommendations for Syndrome Definition and Measurement Techniques in Clinical Research. The Report of an American Academy of Sleep Medicine Task Force. Sleep.

[37] Fietze I., Laharnar N., Obst A., Ewert R., Felix S.B., Garcia C., Gläser S., Glos M., Schmidt C.O., Stubbe B. (2019). Prevalence and association analysis of obstructive sleep apnea with gender and age differences—Results of SHIP-Trend. J. Sleep Res..

[38] Bjørner J.B., Damsgaard M.T., Watt T., Bech P., Rasmussen N.K., Kristensen T.S., Modvig J., Thunedborg K. (1997). Dansk Manual til SF-36: Et Spørgeskema om Helbredsstatus [Danish Manual for SF-36: A Questionnaire about Health Status].

[39] Smith I.E., Shneerson J.M. (1995). Is the SF 36 sensitive to sleep disruption?. A study in subjects with sleep apnoea. J. Sleep Res..

[40] Sil A., Barr G. (2012). Assessment of predictive ability of Epworth scoring in screening of patients with sleep apnoea. J. Laryngol. Otol..

[41] Colt H.G., Haas H., Rich G.B. (1991). Hypoxemia vs sleep fragmentation as cause of excessive daytime sleepiness in obstructive sleep apnea. Chest.

[42] Punjabi N.M., Bandeen-Roche K., Marx J.J., Neubauer D.N., Smith P.L., Schwartz A.R. (2002). The association between daytime sleepiness and sleep-disordered breathing in NREM and REM sleep. Sleep.

[43] Baldwin C.M., Griffith K.A., Nieto F.J., O’Connor G.T., Walsleben J.A., Redline S. (2001). The Association of Sleep-Disordered Breathing and Sleep Symptoms with Quality of Life in the Sleep Heart Health Study. Sleep.

[44] Bulcun E., Ekici A., Ekici M. (2012). Quality of life and metabolic disorders in patients with obstructive sleep apnea. Clin. Investig. Med..

[45] Sampaio R., Pereira M.G., Winck J.C. (2012). Psychological morbidity, illness representations, and quality of life in female and male patients with obstructive sleep apnea syndrome. Psychol. Health Med..

[46] Blanco J., Zamarrón C., Abeleira Pazos M.T., Lamela C., Suarez Quintanilla D. (2005). Prospective evaluation of an oral appliance in the treatment of obstructive sleep apnea syndrome. Sleep Breath.

[47] Marklund M., Carlberg B., Forsgren L., Olsson T., Stenlund H., Franklin K.A. (2015). Oral Appliance Therapy in Patients With Daytime Sleepiness and Snoring or Mild to Moderate Sleep Apnea: A Randomized Clinical Trial. JAMA Intern. Med..

[48] Barnes M., McEvoy R.D., Banks S., Tarquinio N., Murray C.G., Vowles N., Pierce R.J. (2004). Efficacy of Positive Airway Pressure and Oral Appliance in Mild to Moderate Obstructive Sleep Apnea. Am. J. Respir. Crit. Care Med..

[49] Engleman H.M., McDonald J.P., Graham D., Lello G.E., Kingshott R.N., Coleman E.L., Mackey T.W., Douglas N.J. (2002). Randomized Crossover Trial of Two Treatments for Sleep Apnea/Hypopnea Syndrome: Continuous Positive Airway Pressure and Mandibular Repositioning Splint. Am. J. Respir. Crit. Care Med..

